# Surgical management and final outcomes of chondrosarcoma of the temporomandibular joint: case series and comprehensive literature review

**DOI:** 10.1186/s12957-023-03143-1

**Published:** 2023-08-19

**Authors:** Hyun Jun Oh, Hye-Jung Yoon, Kyung-Hoe Huh, Bongju Kim, Ik-Jae Kwon, Soung Min Kim, Joo Yong Park, Sung Weon Choi, Jong-Ho Lee

**Affiliations:** 1 Oral Oncology Clinic, National Cancer Center, Goyang, Korea; 2 Department of Oral Pathology, Dental Research Institute, School of Dentistry, Seoul National University, Seoul, Korea; 3 Department of Oral and Maxillofacial Radiology, Dental Research Institute, School of Dentistry, Seoul National University, Seoul, Korea; 4 Dental Life Science Research Institute, Innovation Research & Support Center for Dental Science, Seoul National University Dental Hospital, Seoul, Korea; 5 Department of Oral and Maxillofacial Surgery, Dental Research Institute, School of Dentistry, Seoul National University, Seoul, Korea

**Keywords:** Chondrosarcoma, Temporomandibular joint, TMJ reconstruction, Facial nerve transection, Nerve graft

## Abstract

**Background:**

Surgical management for chondrosarcoma of the temporomandibular joint (TMJ) is challenging due to the anatomical location involving the facial nerve and the functional joint. The purpose of this case series was to analyze the largest number of TMJ chondrosarcoma cases reported from a single institution and to review the literature about chondrosarcoma involving the TMJ.

**Methods:**

Ten TMJ chondrosarcoma patients at Seoul National University Dental Hospital were included in this study. Radiographic features, surgical approaches, histopathologic subtypes, and treatment modalities were evaluated. All case reports of TMJ chondrosarcoma published in English from 1954 to 2021 were collected under PRISMA guidelines and comprehensively reviewed.

**Results:**

The lesions were surgically resected in all 10 patients with efforts to preserve facial nerve function. Wide excision including margins of normal tissue was performed to ensure adequate resection margins. All TMJs were reconstructed with a metal condyle except one, which was reconstructed with vascularized costal bone. At last follow-up, all patients were still alive, and there had been no recurrence. Among 47 cases (patients from the literature and our cases), recurrence was specified in 43 and occurred in four (9.5%).

**Conclusions:**

For surgical management of TMJ chondrosarcoma, wide excision must consider preservation of the facial nerve. Reconstruction using a metal condyle prosthesis and a vascularized free flap is reliable. A more conservative surgical approach correlates with a favorable prognosis for facial nerve recovery. Nevertheless, wide excision is imperative to prevent tumor recurrence. In cases in which the glenoid fossa is unaffected by the tumor, it is deemed unnecessary to reconstruct the glenoid fossa within an oncological setting.

## Background

Chondrosarcoma is a malignant neoplasm in which the tumor cells form a cartilaginous matrix [1]. It is the second most frequent primary malignant tumor of the bone after osteosarcoma [2]. However, the head and neck region has been reported as a rare site of origin for chondrosarcoma, accounting for 1 to 12% of chondrosarcoma cases [3]. Furthermore, chondrosarcoma in the temporomandibular joint (TMJ) area is extremely rare.

Diagnosing chondrosarcoma in the head and neck region is challenging because cartilaginous neoplasms are rare and exhibit various histopathologic patterns, from benign chondroid tumors to malignant undifferentiated neoplasms [4]. According to previous studies, delays in diagnosing TMJ chondrosarcoma have reached 6 to 8 years [5, 6]. Treating chondrosarcoma of the TMJ is also challenging because of the proximity of vital structures and the consequent difficulty of performing an adequate resection. Cranial invasion has been reported previously [5, 6], and chondrosarcoma of the facial skeleton has been reported to have poorer prognosis than that in other regions of the body [7]. The latest therapeutic concepts in TMJ surgery, including resection guide by digital osteotomy templates [8], pre-bent and milled plates [9], 3D-printed patient-specific prostheses [10], and intraoperative navigation [11], can aid in accurate resection and reconstruction.

During the past 20 years, we have encountered multiple cases of destructive cartilaginous neoplasms in the TMJ and neighboring anatomy that were diagnosed by magnetic resonance imaging (MRI) and open biopsy. This article provides a detailed analysis of that case series and a comprehensive literature review of chondrosarcoma involving the TMJ to evaluate diagnostic approaches, management modalities, and final outcomes.

## Methods

### Subjects

The study sample contains patients with chondrosarcoma arising in the TMJ for which they underwent therapeutic surgery at the Department of Oral and Maxillofacial Surgery at Seoul National University Dental Hospital from April 1, 2000 through October 31, 2021. This study was approved by the Institutional Review Board of Seoul National University Dental Hospital (IRB approval number: ERI21040).

### Treatment protocol

In this study, MRI, contrast-enhanced computed tomography (CT), and positron emission tomography-CT (PET-CT) were performed for radiographic examination [12]. Open biopsy was conducted for pathologic confirmation of the diagnosis. For the definite surgery, wide excision and immediate reconstruction were conducted on the same day. Histopathologically, chondrosarcoma is categorized into three subtypes (grades I, II, and III) according to nuclear size, cellularity, and frequency of mitosis [13]. Post-operative radiation therapy was performed for high-grade cases and cases in which the surgical resection margin was involved in pathology. Considering the possibility of primary site recurrence and lung metastasis, contrast-enhanced CT and chest X-ray were recommended at intervals of 3–6 months.

### Facial nerve approach

Three surgical approaches to the facial nerve were used according to size and location of the tumor. When the facial nerve was involved in the tumor, segmental resection and nerve graft were performed [14, 15]. When the nerve was retracted to remove the tumor and the tension on the nerve increased, intentional cut and end-to-end repair with epineural suture were used [16]. When the tension did not increase during nerve isolation and retraction, the nerve was preserved without intervention.

Facial nerve function was evaluated using the House-Brackmann (HB) grading scale at least 1 year after the surgical procedure [17]. The HB system assigns patients to categories I to VI based on their degree of facial function. Grade I is normal function, and grade VI is total paralysis.

### TMJ reconstruction

The resected condylar and mandibular areas were reconstructed using vascularized costal bone, a condylar prosthesis with a reconstruction plate (Stryker, Kalamazoo, MI, USA), or a ramal condylar implant (Zimmer Biomet, Jacksonville, FL, USA) [18, 19]. To prevent soft tissue depression in the pre-auricular area and to fill the tumor defect, a vascularized serratus anterior or latissimus dorsi free flap was transferred with microvascular anastomosis [20]. Reconstruction of the glenoid fossa depended on the surgical resection margin. When the glenoid fossa was included in the surgical resection margin clinically or pathologically by intraoperative frozen biopsy, the fossa was reconstructed using a fossa implant (Zimmer Biomet, Jacksonville, FL, USA) [19].

### Literature search

**Fig. 1:**
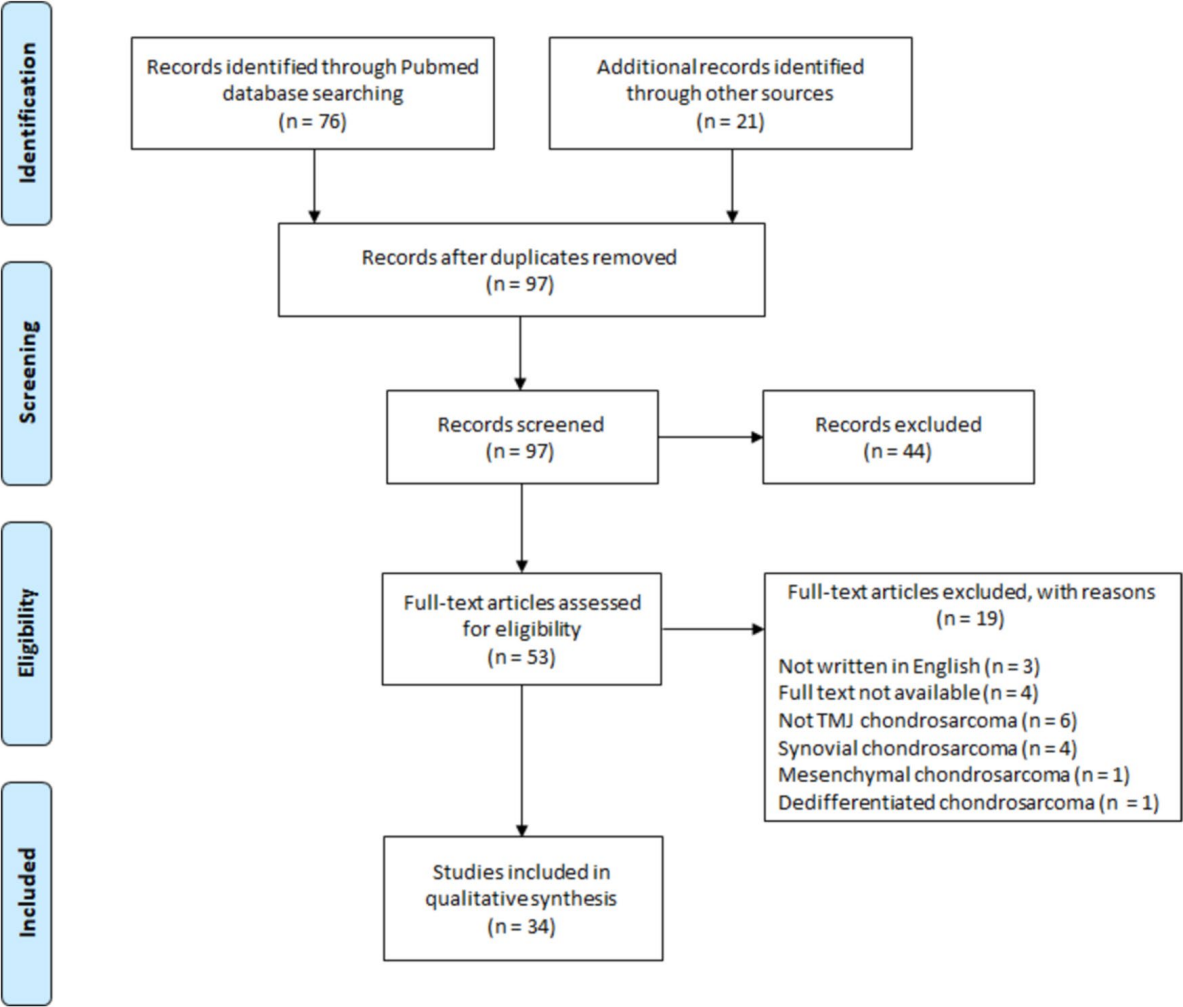
PRISMA flow chart of systematic search on TMJ chondrosarcoma (*PRISMA* Preferred Reporting Items for Systematic reviews and Meta‑Analyses, *TMJ* Temporomandibular joint)

This study followed the Preferred Reporting Items for Systematic Reviews and Meta-Analyses (PRISMA) guidelines [21] (Fig. 1). All case reports about TMJ chondrosarcoma published in English from 1954 to 2021 were collected. The literature search was carried out in PubMed using the keywords “chondrosarcoma” and “temporomandibular joint.” In addition, the reference lists of the retrieved articles were manually cross-checked.

### Eligibility criteria and data analysis

All publications reporting cases of chondrosarcoma arising in the TMJ were eligible. The inclusion criterion was conventional chondrosarcoma cases. Other types, including synovial, mesenchymal, and dedifferentiated chondrosarcomas, were excluded. Articles that were not written in English or whose entire text was unavailable were also excluded. Each individual case was thoroughly reviewed to obtain data on demographics, diagnosis, tumor extent, surgical management, adjunctive therapy, and treatment outcomes.

## Results

### Radiographic findings

**Fig. 2:**
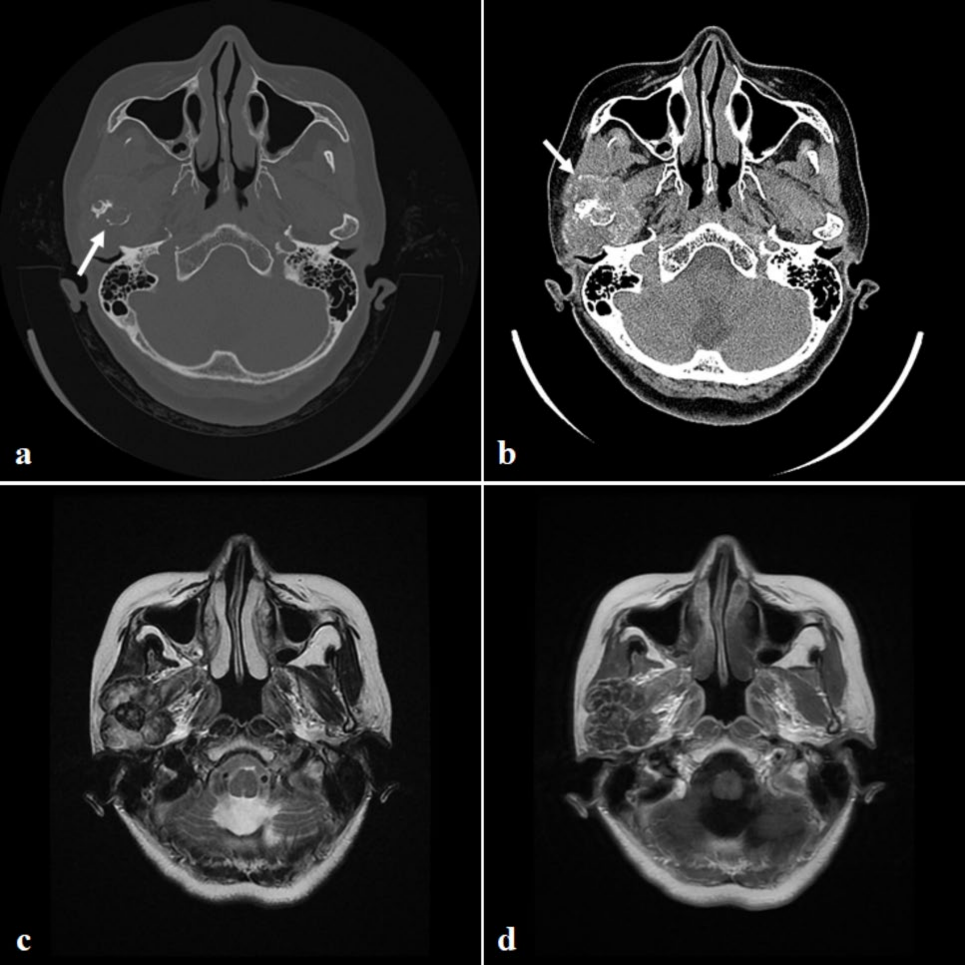
Pre-operative images (case 2) **a** CT (bone window image): an irregularly destructed condyle is observed. The cortical bone of the condyle is severely perforated and bone marrow is also infiltrated. **b** CT (soft tissue window image): tiny calcified foci are scattered around the margin of and inside the mass. **c** MRI (T2 weighted image): heterogeneous high signal intensity is observed around the right condyle. **d** MRI (contrast-enhanced T1 weighted image): heterogeneous enhancement around the right condyle is noted

The following radiological findings were observed in MRI and CT images: condyle destruction, sclerotic changes in the condyle, displacement of surrounding structures by a mass in the TMJ, calcified foci inside the mass, bulging of the mass with an enhancing rim, and joint space widening (Fig. 2). In this case series, distant metastasis was not found on pre-operative PET-CT in any cases.

### Facial nerve recovery

**Fig. 3:**
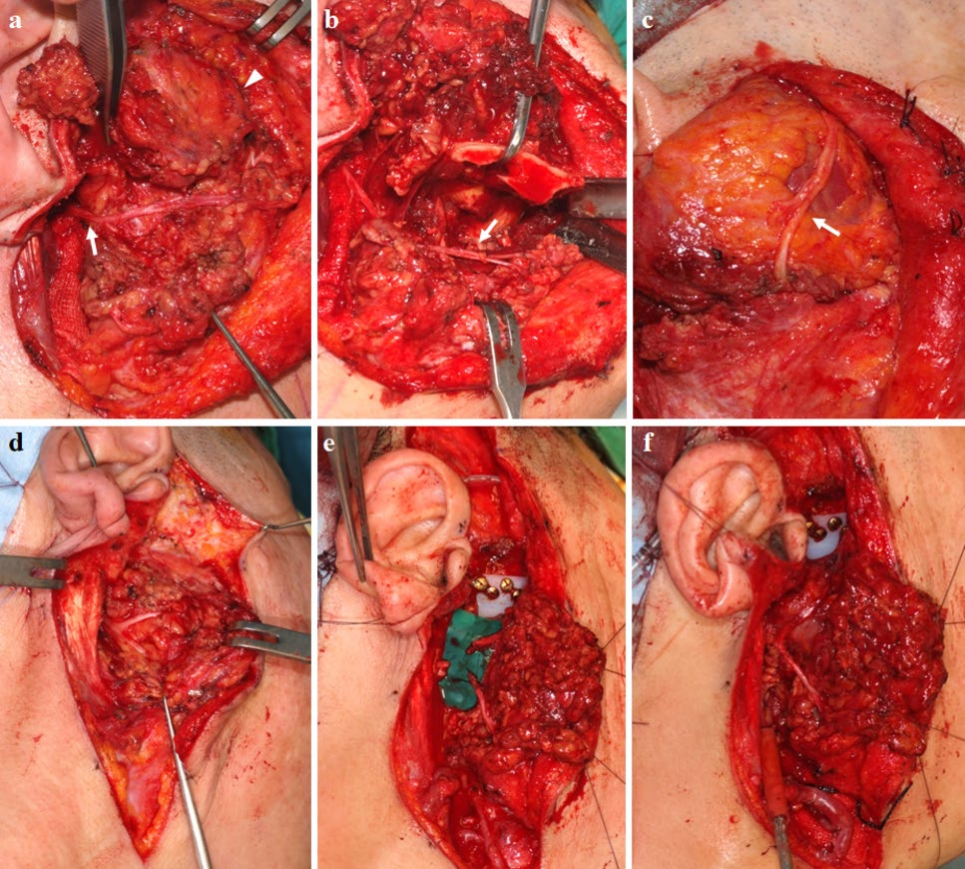
Surgical approaches to the facial nerve **a** (Case 2) Segmentally resected temporofacial trunk (arrowhead) and preserved cervicofacial trunk (arrow). **b** (Case 2) Retracted buccal branch (arrow) during mass resection. **c** (Case 2) Nerve graft using 10 cm length of thoracodorsal nerve for temporofacial trunk (arrow). **d** (Case 4) Temporofacial and cervicofacial trunks before intentional cut. **e** (Case 4) Intentionally cut temporofacial and cervicofacial trunks. **f** (Case 4) Temporofacial and cervicofacial trunks after end-to-end nerve repair

**Fig. 4:**
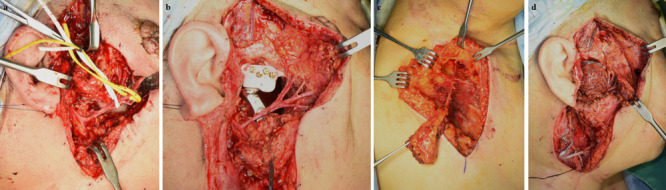
Reconstruction of the mandibular condyle and glenoid fossa and surgical approach to the facial nerve (Case 7) **a** Facial nerve isolation and retraction during mass resection. **b** Preserved facial nerve after mass resection and reconstruction with a fossa implant and a metal condyle prosthesis. **c** Harvesting of the serratus anterior free flap with a vascular pedicle (the serratus branch of the thoracodorsal artery and the vena comitans). **d** Reconstruction with micro-anastomosed serratus anterior free flap

Of the 10 cases in our institution, segmental resection was performed in two because of nerve involvement in the tumor (Table 1 and Fig. 3). In one case, a segment of the frontal branch of the facial nerve was resected, and the great auricular nerve was grafted interpositionally (case 1). In the other, the temporofacial trunk was partially resected and interpositionally grafted with the thoracodorsal nerve from a latissimus dorsi free flap (case 2). In four cases, the facial nerve was intentionally cut due to high tension while retracting it during approach to the mass (Fig. 3). The zygomatic branch (case 3), temporofacial trunk (case 9), and temporofacial and cervicofacial trunks (cases 4 and 5) of the facial nerve were intentionally cut, and neurorrhaphy was conducted. In the other four cases, the facial nerve was preserved because no excessive retraction was required during the tumor approach (Fig. 4). In cases involving segmental resection, the patients experienced moderately severe facial nerve dysfunction (HB grade IV). When an intentional cut was performed, facial nerve dysfunction was moderate-mild (HB grade III) or mild (HB grade II) at more than 1 year after surgery. In the patients who underwent only nerve retraction, nerve dysfunction was mild (HB grade II) or absent (HB grade I).

**Table 1:** Reconstruction of the TMJ after chondrosarcoma ablation and surgical approaches to the facial nerve in our cases

Case	Mandibular condyle reconstruction	Glenoid fossa reconstruction	Free flap for soft tissue reconstruction	Facial nerve approach	Resected or cut nerve area	Nerve repair method (grafted nerve)	Post-operative House-Brackmann grade ^a^
1	Vascularized costal bone	None	LD & SA	Segmental resection	Frontal branch	Nerve graft (great auricular nerve)	No data
2	Condylar prosthesis and R-plate	None	LD	Segmental resection	Temporofacial trunk	Nerve graft (thoracodorsal nerve)	IV
3	Condylar prosthesis and R-plate	None	SA	Intentional cut	Zygomatic branch	End to end repair	II
4	Condylar prosthesis and R-plate	Fossa implant	SA	Intentional cut	Temporofacial trunk, Cervicofacial trunk	End to end repair	III
5	Condylar prosthesis and R-plate	Fossa implant	LD	Intentional cut	Temporofacial trunk, Cervicofacial trunk	End to end repair	III
6	Condylar prosthesis and R-plate ^b^	None	LD	Retraction	None	None	II
7	Ramal condylar implant	Fossa implant	SA	Retraction	None	None	II
8	Ramal condylar implant	Fossa implant	SA	Retraction	None	None	I
9	Ramal condylar implant	Fossa implant	SA	Intentional cut	Temporofacial trunk	End to end repair	Follow-up loss
10	Condylar prosthesis and R-plate	Fossa implant	SA	Retraction	None	None	I

House-Brackmann grading scale: *IV* moderately severe dysfunction, *III* moderate dysfunction, *II* mild dysfunction, *I* normal

*R-plate* reconstruction-plate, *LD* latissimus dorsi, *SA* serratus anterior

^a^Post-operative House-Brackmann grading scale was evaluated at least one year after the surgical procedure

^b^The R-plate fractured 23 months after the primary surgery and the mandible was reconstructed with a vascularized fibular free flap

### Mandibular condyle and glenoid fossa reconstruction

**Fig. 5:**
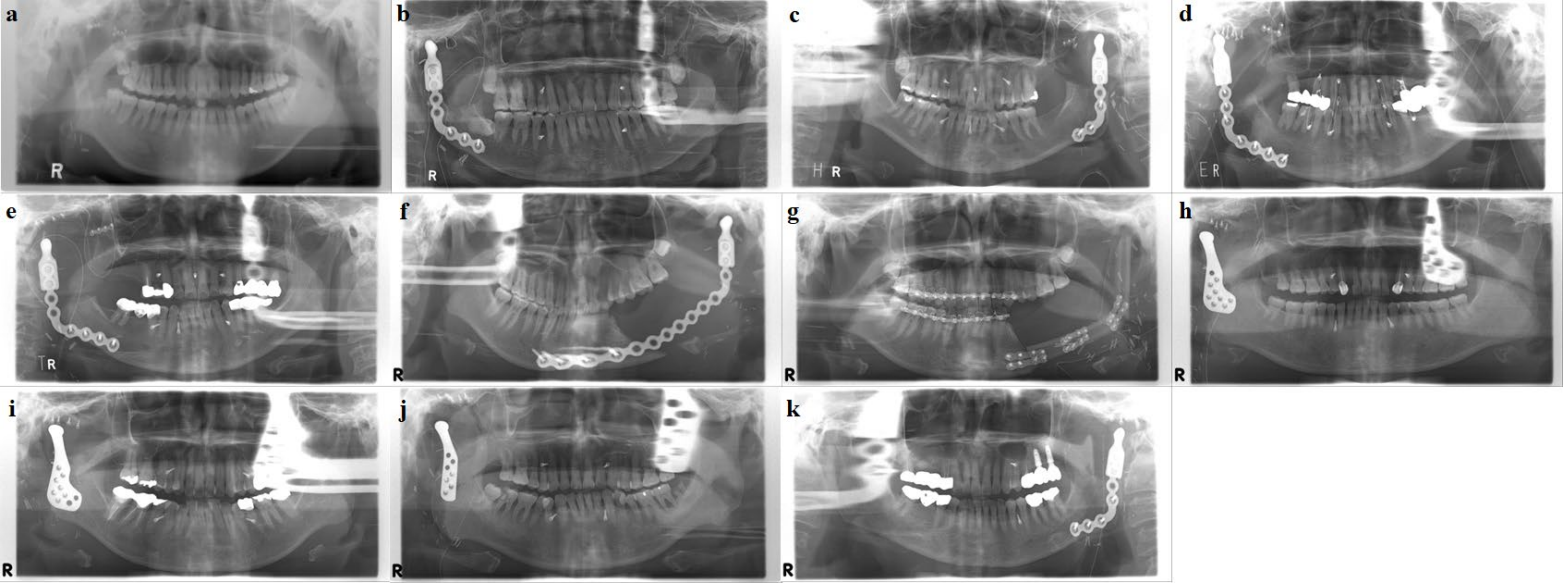
Reconstruction of the mandibular condyle and glenoid fossa, post-operative panoramic radiographs **a** (Case 1) The condyle was reconstructed with vascularized costal bone. **b** (Case 2) The condyle was reconstructed with a condylar prosthesis with a reconstruction plate. **c** (Case 3) The condyle was reconstructed with a condylar prosthesis with a reconstruction plate. **d** (Case 4) The condyle was reconstructed with a condylar prosthesis with a reconstruction plate and the glenoid fossa was reconstructed with a fossa implant. **e** (Case 5) The condyle was reconstructed with a condylar prosthesis with a reconstruction plate and the glenoid fossa was reconstructed with a fossa implant. **f** (Case 6) The condyle was reconstructed with a condylar prosthesis with a reconstruction plate. **g** (Case 6) The reconstruction plate fractured 23 months after the primary surgery and the condylar and mandibular areas were reconstructed with a vascularized fibular free flap. **h** (Case 7) The condyle was reconstructed with a metal condyle prosthesis and the glenoid fossa was reconstructed with a fossa implant. **i** (Case 8) The condyle was reconstructed with a metal condyle prosthesis and the glenoid fossa was reconstructed with a fossa implant. **j** (Case 9) The condyle was reconstructed with a metal condyle prosthesis and the glenoid fossa was reconstructed with a fossa implant. **k** (Case 10) The condyle was reconstructed with a condylar prosthesis with a reconstruction plate and the glenoid fossa was reconstructed with a fossa implant

All patients underwent primary mass removal and reconstructive surgery simultaneously. The resected condyle area was reconstructed with a condylar prosthesis with a reconstruction plate or ramal condylar implant except in one patient, whose TMJ was reconstructed with vascularized costal bone (Table 1 and Fig. 5). In six patients, the glenoid fossa was also reconstructed using a fossa implant (Figs. 4 and 5). In case 6, the reconstruction plate fractured 23 months after the primary surgery, and the mandible was reconstructed with a fibular free flap (Fig. 5).

### Histopathologic findings

**Fig. 6:**
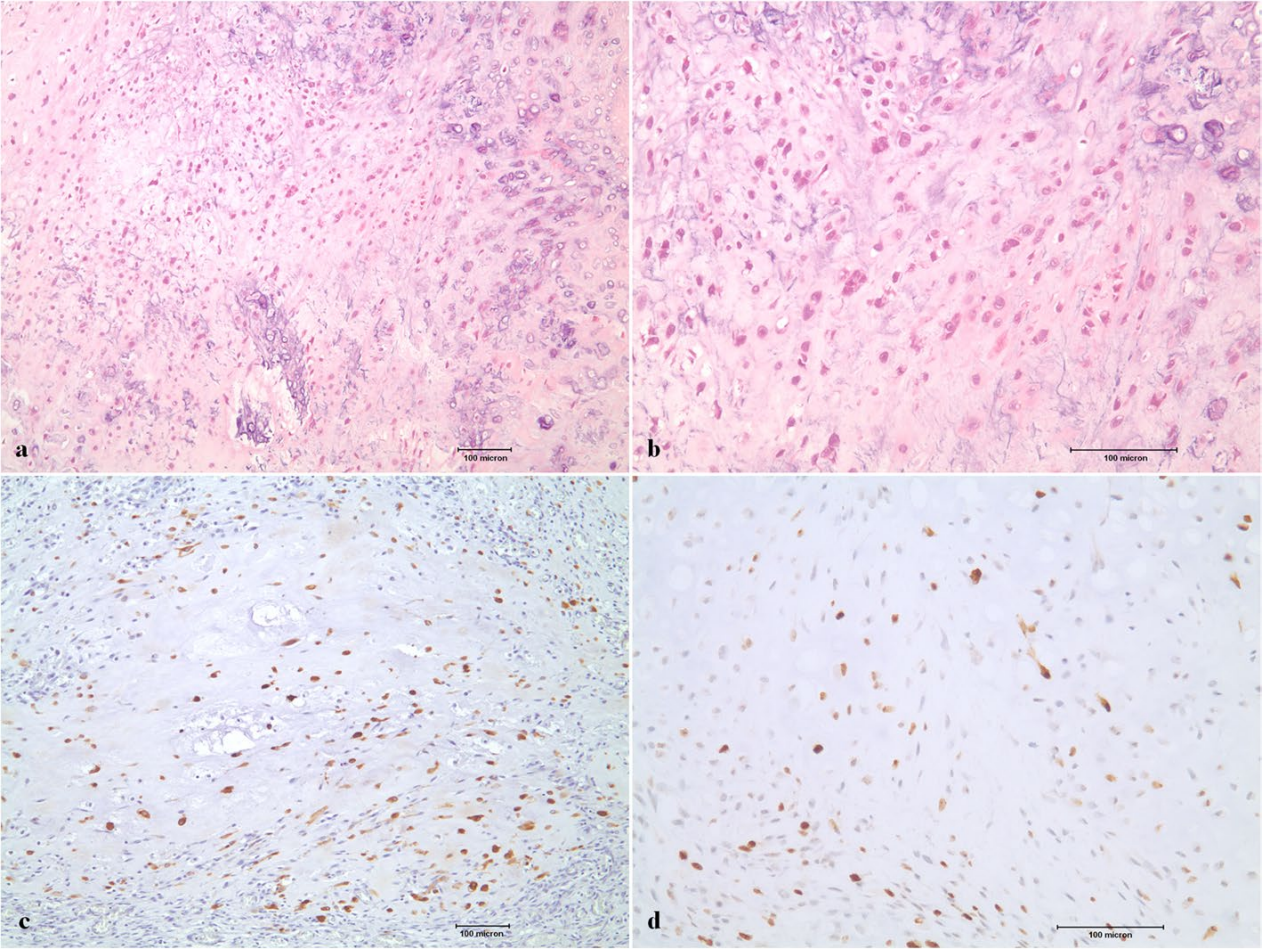
Histopathologic images **a** H&E staining, × 100 (case 7, grade II). The lesion showed proliferation of tumor cells within the chondroid matrix with focal calcification. **b** H&E staining, × 200 (case 7, grade II) Atypical chondrocytes presented with increased cellularity, pleomorphism, multinucleation, and mitoses. **c** S-100 protein, × 100 (case 3, grade II) immunohistochemistry showed diffuse positive expression of S-100 protein. **d** Ki-67, × 200 (case 4, grade II) immunohistochemistry showed 15–20% positive expression of Ki-67

Microscopic examination of sections stained with hematoxylin–eosin showed atypical chondrocytes exhibiting increased cellularity, pleomorphism, multinucleation, and mitoses. Chondrosarcomas demonstrate proliferation of tumor cells within the chondroid matrix with focal calcification, as seen in Fig. 6. On stained slides, we identified surgical resection margins by measuring distances from the tumor borders in the anterior, posterior, superior, inferior, medial, and lateral directions. When tumor cells were observed in the margins in more than one direction, the sample was judged to have positive margins. Immunohistochemical examination was carried out with antibodies against vimentin, S-100 protein, and Ki-67. All cases were positive for vimentin and showed diffuse positive expression of S-100 protein. Ki-67 was expressed in more than 25% of grade III, 10–20% of grade II, and less than 5% of grade I patients (Fig. 6).

### Post-operative courses

Seven patients received post-operative radiation therapy (Table 2) and experienced complications such as pain, trismus, facial nerve weakness, headache and amblyacousia, for which they underwent rehabilitation treatment such as physical therapy through the department of rehabilitation medicine. The mouth openings for most cases were greater than 25 mm. However, the mouth openings for cases 1 and 7 were 18 mm and 22 mm, respectively. In case 5, the patient’s mouth opening was sufficient to allow dental implant surgery under local anesthesia about 21 months after the primary operation. In case 1, grafted costal bone in the ramus area was partially removed due to an infection at 2 months post-operatively. No other post-operative infections occurred. All patients were alive at last follow-up, and no recurrences in the condyle or glenoid fossa had occurred. In case 6, lung metastasis occurred 16 months after the mass resection surgery. After partial lobectomy, the patient is undergoing periodic follow-up.

**Table 2:** Summary of TMJ chondrosarcoma cases in the literature and our series

Study	Year	Age	Sex	Tumor size (cm)	Facial nerve preservation^a^	Glenoid fossa reconstruction	Histopathologic grade	PORT	Follow up period (month)	Recurrence (time to recur)
Nortje [22]	1976	40	M	6.0	—	N	I	N	24	N
Looser [23]	1976	26	M	4.0	—	—	—	N	54	Y^b^ (12 months)
Morris [24]	1987	29	F	2.5	Y	—	I	Y	6	N
Murayama [25]	1988	30	M	8.0 × 9.5 × 10.0	—	—	II	Y^c^	28	N
Wasenko [26]	1990	49	F	4.0 × 2.5	—	—	I	N	—	—
Park [27]	1992	25	M	—	—	—	—	N	11	N
Nitzan [5]	1993	36	F	2.5	N	—	I	N	84	N
Sesenna [7]	1997	60	F	—	—	—	I	N	60	N
Blanchaert [28]	1998	42	M	—	Y	—	II	Y	43	Y (5 months)
Batra [29]	1999	65	M	4.0 × 1.8	Y	—	I	N	7	N
Moustafapour [6]	2000	23	F	6.0 × 6.0	—	—	I	N	—	—
Moustafapour [6]	2000	52	F	6.0 × 4.0 × 3.0	—	—	I	N^d^	12	Y^e^ (12 months)
Sasaki [30]	2002	45	F	—	—	—	—	N	72	N
Yun [31]	2008	29	F	1.5 × 1.3 × 1.1	—	—	I	N	—	—
Acar [32]	2008	32	M	2.5 × 3.0	—	—	I	Y	48	N
Acar [32]	2008	40	F	2.5 × 1.0 × 1.0	—	—	I	Y	43	N
Gallego [33]	2009	54	M	2.2 × 1.3 × 0.5	Y	—	I	N	16	N
Oliveira [34]	2009	11	F	2.7 × 1.9	—	—	I	N	72	N
Garzino-Demo [35]	2010	65	F	—	Y	N	I	Y	108	N
Gonzalez-Perez [36]	2011	57	M	—	—	Y	I	N	24	N
Xu [37]	2011	34	F	8.0 × 6.0 × 5.0	—	—	I	N	—	—
Deyhimi [38]	2012	63	F	4.0 × 3.0 × 2.0	—	—	II	N	—	—
Ramos-Murguialday [39]	2012	45	M	5.5 × 2.0	—	N	II	N	36	N
Abu-Serriah [40]	2013	48	M	1.0	—	Y	II	N	6	N
Goutzanis [41]	2013	23	M	6.0	Y	N	I–II	N	24	N
Kumar Reddy [42]	2014	7	M	5.0 × 3.0	—	N	I	N	12	N
Giorgione [43]	2015	56	M	4.6 × 4.0 × 3.9	Y	—	I	Y	—	N
MacIntosh [44]	2015	31	F	3.0	—	Y	I	N	336	Y (123 months)
Nomura [45]	2015	28	M	4.0 × 5.0 × 3.0	—	N	I	N	120	N
Lee [46]	2016	47	F	5.0 × 3.5 × 3.0	N	N	I	Y	47	N
Fukada [47]	2018	78	F	4.0 × 3.6	Y	N	II	N	84	N
Inomata [48]	2020	42	M	4.0 × 3.5 × 3.0	Y	N	I	N	84	N
Le [49]	2020	47	F	2.0 × 2.0	Y	—	I	Y	6	N
Ampu [50]	2021	70	F	3.1 × 3.5 × 2.3	—	N	I	N	15	N
Iro [51]	2021	54	M	2.5 × 3.3 × 4.3	Y	—	I–II	Y	48	N
Iro [51]	2021	61	F	—	—	—	—	Y	12	N
Chia [52]	2021	67	M	4.0 × 2.9 × 2.5	N	—	II–III	Y	18	N
Present author	2023	19	M	4.0 × 3.0 × 3.0	N	N	I	N	196	N
Present author	2023	54	F	3.5 × 3.0 × 2.5	N	N	I	N	134	N
Present author	2023	44	F	3.0 × 2.5 × 1.8	N	N	II	Y	86	N
Present author	2023	62	F	3.8 × 3.0 × 3.0	N	Y	II	N	85	N
Present author	2023	64	F	5.0 × 4.7 × 3.6	N	Y	I	Y	87	N
Present author	2023	16	F	5.1 × 3.9 × 2.8	Y	N	III	Y	66	N^f^
Present author	2023	49	F	1.3 × 2.8 × 2.5	Y	Y	II	Y	36	N
Present author	2023	51	F	2.6 × 2.9 × 1.7	Y	Y	I	Y	25	N
Present author	2023	55	M	3.2 × 2.5 × 2.6	N	Y	II	Y	1	N
Present author	2023	64	F	2.3 × 1.4 × 2.5	Y	Y	III	Y	12	N

*M* male, *F* female, *PORT* post-operative radiation therapy, *Y* yes, *N* no, — not specified)

^a^This pertains to cases in which the continuity of the facial nerve was disrupted irrespective of nerve repair

^b^The patient died of massive recurrent disease

^c^Concurrent chemo-radiation therapy was performed

^d^PORT recommended due to positive margins but declined by the patient

^e^The patient returned one year later with recurrence and underwent RT

^f^Lung metastasis occurred 16 months after the resection surgery. After partial lobectomy, the patient is undergoing periodic follow-up

### Literature review

The 34 articles reporting on 37 cases on chondrosarcoma of the TMJ published between 1976 and 2021 are summarized in Table 2 [5–7, 22–46, 48–52]. Of the 37 cases, facial nerve preservation was reported in 14, and the facial nerve was sacrificed in 3 cases. Glenoid fossa reconstruction was reported in 13 cases, and glenoid fossa reconstruction was completed for 3 patients. Among the 47 total cases, including our cases, the tumor grade was specified in 43. Among them, 28 cases (65.1%) were reported to be grade I, 2 (4.7%) were grade I–II, 10 (23.3%) were grade II, 1 (2.3%) was grade II–III, and 2 (4.7%) were grade III. All the patients underwent surgical therapy. Of the 47 patients, 19 underwent post-operative radiation therapy. Among the 42 cases in which recurrence was specified, it occurred in 4 (9.5%). Of the 4 cases with recurrence, 3 recurred within 1 year, and the other recurred after 10 years. Among those 4 patients, 1 died of the recurred disease, and the other 3 survived. Distant metastasis to the lung occurred in 1 patient who remained alive after partial lobectomy.

## Discussion

Chondrosarcoma arising in the TMJ area is extremely rare. Among the 10 patients in our series, 2 were male and 8 were female (Table 2). This female predominance is consistent with previous studies [46, 47, 53]. The mean age of the patients in our study was 48.3 (16–64) years. This was not different from recent studies, in which the reported mean ages were 46.7 years [46] and 46.5 years [47, 53].

The major radiological features of chondrosarcoma arising in the TMJ were consistent with previous studies [35, 39, 45–47, 53]. MRI is recommended for preoperative planning because it provides detailed information about the anatomic limits and the most accurate assessment of the extent of the lesion [34, 36]. Contrast-enhanced CT is also recommended. In our cases and the cases from the literature, CT and MRI demonstrated bony destruction and tumor spread, which aided in the differential diagnosis and enabled creation of an adequate treatment plan [24, 45]. Image findings such as outward growth from the condyle, various patterns of internal enhancement, and markedly hyperintense T2 signal areas were noted [12]. In our cases, an active lesion in the TMJ with no metastatic bone lesion was observed on PET-CT.

Biopsy was performed for diagnosis and pre-operative confirmation of chondrosarcoma. Biopsy of chondrosarcoma is not recommended by some authors because of the risk of anaplastic transformation and diffusion during manipulation of the mass [54, 55]. Moreover, fine needle aspiration biopsy does not always provide a reliable diagnosis [6, 24, 36, 53]. Nonetheless, incisional biopsy is the best option for distinguishing among osteogenic sarcoma, pleomorphic adenoma, and chondroma [35]. Other authors recommend open biopsy as a definite means to achieve final diagnosis [45]. In our cases, aspiration biopsy and core needle biopsy were ineffective. In case 5, aspiration biopsy failed, and the result of a subsequent core needle biopsy under local anesthesia performed 4 years later was a hyperplastic cartilage chip. However, an incisional biopsy under general anesthesia diagnosed chondrosarcoma 1 month after the previous biopsy. In case 7, the result of a core needle biopsy under local anesthesia was fibromuscular tissue with no diagnostic abnormalities. However, the result of an open biopsy performed 2 months later under general anesthesia was chondrosarcoma. Core needle biopsy has been recognized as a dependable diagnostic technique for musculoskeletal tumors, exhibiting minimal associated complications [56, 57]. However, core needle biopsy was not conducted in previous cases of TMJ chondrosarcoma and yielded unsatisfactory results in cases 5 and 7 among our patients. Incorporation of ultrasound [58], CT [59], or computer-assisted navigation [60] alongside core needle biopsy might enhance its diagnostic capabilities for TMJ diseases.

Surgical management is the standard primary treatment for chondrosarcoma [61–66]. Wide local resection was performed in all our cases. No significant cervical lymphadenopathy was observed in any cases. Selective neck dissection was performed only in case 1. No metastasis to regional lymph nodes was observed in the biopsy. This is consistent with previous studies, which concluded that neck node dissection is usually unnecessary because of the low incidence of regional lymph node metastasis [3, 65]. In our cases, the treatment protocol did not include neck dissection unless suspicious cervical lymph nodes were found. After successful tumor resection, facial appearance is an important outcome in the head and neck area. A vascularized free flap was used for reconstruction in our cases. In the literature, local flaps such as a temporal muscle flap [5, 35] or microvascular free flaps such as a fibular free flap [39, 46] have been used depending on the defect size and location. A serratus anterior free flap for relatively small defects and a latissimus dorsi free flap for relatively large defects were used in our cases. Costal bone was also harvested with the serratus anterior muscle for reconstruction of the mandibular condyle and ramus (case 1) or the zygomatic process of the maxilla (case 3).

Among the post-operative complications, facial nerve weakness was thought to be due to segmental resection or an intentional nerve cut when those approaches were used or due to irritation during the operation. Facial nerve function gradually recovered through rehabilitation treatment such as physical therapy. With respect to post-operative facial nerve function, the HB grades were closest to normal in cases in which the nerve was not transected (Table 1). Patients were able to close their eyes completely in all cases except those involving segmental resection. Prognosis was better in cases involving an intentional cut than in those involving segmental resection, and prognosis was also better when one of the five branches of the facial nerve was transected than when the temporofacial or cervicofacial trunk was transected. Although a conservative approach to the facial nerve yielded better recovery of nerve function, a radical approach might be required to completely resect the mass and prevent recurrence.

Because the patient in case 1 was only 19 years old, the mandibular condyle was reconstructed with vascularized costal bone instead of an artificial prosthesis. However, ankylosis of the TMJ occurred during follow up. Mouth opening improved after gap arthroplasty. Although a costochondral graft is a viable option with growth potential, recurrent ankylosis has been reported as a major problem [67, 68]. After ablative surgery, joint ankylosis has been reported when a vascularized fibular free flap is used to reconstruct the mandibular condyle [69]. When autogenous bone is being considered for condyle reconstruction, the possibility of ankylosis should be considered. In the present study, condyles were reconstructed using a condylar prosthesis with no fossa reconstruction in 3 cases (cases 2, 3, and 6). In other studies involving metal condylar replacement alone, the condyle eroded into the glenoid fossa [70] or penetrated the middle cranial fossa [71]. However, Marx et al. reported long-term stability of a metal condyle replacement without alloplastic replacement of the glenoid fossa [18]. The condylar prostheses in the present study were maintained without severe complications. Because the forces imposed on the glenoid fossa by the condylar prosthesis can be reduced in ablative surgery cases, the condylar prostheses seemed to function in place. In case 6, the reconstruction plate fractured 23 months after the primary surgery [72] and was reconstructed with a vascularized fibular free flap. This might have occurred because the boundary of resection in that case was the widest of the 10 cases, leading to a mechanically unfavorable situation.

Chemotherapy was not performed in our cases because the consulting opinion from the department of hemato-oncology was that it would offer no benefit because chondrosarcomas are poorly responsive to chemotherapy. In the literature, chemotherapy was reported to have a limited role in chondrosarcoma treatment, but it can be used as an adjuvant therapy in cases with aggressive behavior, rapid local recurrence, or high-grade chondrosarcoma [3, 33]. Obtaining adequate resection margins with chondrosarcoma arising in the TMJ is challenging because of the proximity to vital structures. Therefore, post-operative radiation therapy was performed in the 7 of our 10 cases that involved surgical resection margin or high-grade chondrosarcoma on pathology. Radiation therapy is recommended post-operatively only for high-grade or incompletely resected chondrosarcomas [73–77]. A treatment protocol involving radical surgery with radiotherapy resulted in a better prognosis in a recent study [65]. In the present cases series, post-operative radiation therapy was also thought to be beneficial because no local recurrence occurred in the 7 applicable patients. However, complications such as trismus, fibrosis, oral mucositis, and xerostomia were observed in those patients. Therefore, radiotherapy should be strictly applied for limited indications.

After the definitive surgeries, periodic follow-up with chest X-ray and enhanced CT is important. In case 6, which involved grade III chondrosarcoma, lung metastasis was found on a chest X-ray 16 months after surgery. Additional imaging with chest CT and PET-CT revealed a right lower lobe mass and no other distant metastases. The patient underwent partial lobectomy and is now undergoing periodic follow-up. Strict observation is ongoing because the recurrence and metastasis occurred after a long period of follow-up [65]. No other distant metastases and no local recurrences were observed. Because of the possibility of primary site recurrence and lung metastasis, it is recommended that enhanced head and neck CTs and chest X-rays be obtained at intervals of 3–6 months.

Histopathologically, chondrosarcoma of the TMJ appears similar to chondrosarcoma of the head and neck or of other regions of the body [33, 46]. In our study, microscopic examination of sections stained with hematoxylin–eosin showed atypical chondrocytes exhibiting increased cellularity, pleomorphism, multinucleation and mitoses. Chondrosarcomas demonstrate proliferation of tumor cells within the chondroid matrix with focal calcification, as seen in Fig. 6. Chondrosarcoma is categorized into three subtypes (grades I, II, and III) according to nuclear size, cellularity, and frequency of mitosis [13]. Low-grade (grade I) chondrosarcoma has occasional binucleated cells and can show atypical cells, which can also be binucleated. Intermediate-grade (grade II) chondrosarcoma presents with a higher cellular population than grade I, with greater degree of nuclear atypia, hyperchromasia and nuclear size. The mitotic rate is low. High-grade (grade III) chondrosarcoma has significant areas of marked pleomorphism; large cells with nuclei that are more hyperchromatic, denser, and larger in size than those in grade II chondrosarcoma; occasional giant cells; and abundant necrosis. The mitotic rate is higher than in grade II chondrosarcoma [13].

The clinical behavior of chondrosarcoma is linked to its histopathologic grade [7]. The 5-year survival rates for grades I, II, and III chondrosarcomas were 90%, 81%, and 43% respectively. The 10-year survival rates were more variable, at 83%, 64%, and 29%, respectively. Metastasis was not observed in grade I chondrosarcoma, but it did occur in 10% of grade II chondrosarcomas and 71% of grade III chondrosarcomas [13]. The most common cause of death in chondrosarcoma is recurrence, not metastasis [33].

In a recent cohort study, the recurrence rate of chondrosarcoma in all parts of the body was 19%, and the recurrence rate of chondrosarcoma in the head and neck was 5% [78]. In the extremities, 45.7%, 34.3%, and 20%, of chondrosarcomas were grades I, II, and III, respectively. By grade, the recurrence rates of chondrosarcoma of the extremities were 11% (grade I), 12% (grade II), and 17% (grade III). In chondrosarcoma of the TMJ, the recurrence rate is lower than in chondrosarcomas of other areas of the body because most cases are low-grade, and relatively few cases with long-term follow-up have been reported. No definitive relationship has been found between histopathologic grade and local recurrence because such recurrence depends primarily on the adequacy of surgical therapy rather than tumor grade [47]. The most important factor in prognosis is surgical resectability [46, 66].

In our cases, immunohistochemical examination was carried out with antibodies against vimentin, S-100 protein, and Ki-67, for which most patients were positive (Fig. 6). Prior to this study, only 7 case reports of TMJ chondrosarcoma included immunostaining [7, 33, 34, 36, 43, 53, 79]. Excluding 1 case [53] that was included in the present study (case 5), S-100 was expressed in 5–35% of chondrocytes [7, 33, 36], and Ki-67 was expressed in 5–50% [33, 36, 43]. Immunohistochemical examination in the present study showed high expression of vimentin in all cases, and diffuse expression of S-100. Ki-67 was expressed in fewer than 5% of grade I chondrosarcomas, 10–20% of grade II chondrosarcomas, and more than 25% of grade III chondrosarcomas. This result was consistent with those in previous studies. Future immunohistochemical investigations of chondrosarcoma are necessary to achieve more accurate diagnoses.

This study is subject to limitations caused by the rarity of TMJ chondrosarcoma, which results in an insufficient number of cases for a robust statistical analysis. Among the 43 cases in which tumor grade was specified, a mere 3 cases were classified as high grade. Recurrence was reported in only 4 cases. Furthermore, the scarcity of cases with long-term follow-up necessitates caution in interpretation. Among the 47 cases included in this analysis, 41 had specified follow-up periods. Of these, only 15 cases were followed for more than 5 years. Notably, 1 case experienced recurrence 10 years post-surgery, although only 4 cases were observed for more than 10 years. Consequently, it is imperative to exercise caution when interpreting the findings of this study.

## Conclusions

To diagnose chondrosarcoma arising in the TMJ, it is recommended to conduct a radiographic assessment comprising MRI, contrast-enhanced CT, and PET-CT, followed by confirmation through histopathological analysis via open biopsy. In terms of treatment, a wide surgical resection is necessary while taking care to the preserve the facial nerve. A conservative approach to the facial nerve is advantageous for optimal recovery outcomes. Nevertheless, a radical approach can be imperative to achieve complete mass excision. Reliable reconstructive surgery can be accomplished using a metal condyle prosthesis in conjunction with a vascularized free flap. If the tumor involves the glenoid fossa of the temporal bone or the temporomandibular joint disc, reconstruction of the glenoid fossa with a fossa implant is necessary. However, in an oncological context, glenoid fossa reconstruction is deemed unnecessary if those structures are unaffected by the tumor. Post-operative radiation therapy is required in cases involving surgical resection margin or when dealing with high-grade chondrosarcoma.

## Data Availability

The datasets used and/or analyzed during the current study are available from the corresponding author upon reasonable request.

## Abbreviations

TMJ: Temporomandibular joint
MRI: Magnetic resonance imaging
HB: House-Brackmann
CT: Computed tomography
PET-CT: Positron emission tomography-computed tomography

## References

[1] Faro TF, Martins-de-Barros AV, Lima G, Raposo AP, Borges MA, Araujo F, Carvalho MV, Nogueira EFC, Laureano Filho JR (2021). Chondros**arcoma of the temporomandibular joint: systematic review and survival analysis of cases reported to date**. Head Neck Pathol.

[2] Lee FY, Mankin HJ, Fondren G, Gebhardt MC, Springfield DS, Rosenberg AE, Jennings LC (1999). Chondrosarcoma of bone: an assessment of outcome. J Bone Joint Surg Am.

[3] Koch BB, Karnell LH, Hoffman HT, Apostolakis LW, Robinson RA, Zhen W, Menck HR (2000). National cancer database report on chondrosarcoma of the head and neck. Head Neck.

[4] Quevedo FC, Quevedo FB, Neto JCB, Ferreira EN, Carraro DM, Soares FA (2017). Case report: Chondrosarcoma of the head and neck. Human Pathology: Case Reports.

[5] Nitzan DW, Marmary Y, Hasson O, Elidan J (1993). Chondrosarcoma arising in the temporomandibular joint: a case report and literature review. J Oral Maxillofac Surg.

[6] Mostafapour SP, Futran ND (2000). Tumors and tumorous masses presenting as temporomandibular joint syndrome. Otolaryngol Head Neck Surg.

[7] Sesenna E, Tullio A, Ferrari S (1997). Chondrosarcoma of the temporomandibular joint: a case report and review of the literature. J Oral Maxillofac Surg.

[8] Ye ZX, Yang C, Chen MJ, Huang D, Abdelrehem A (2015). Digital resection and reconstruction of TMJ synovial chondrosarcoma involving the skull base: report of a case. Int J Clin Exp Med.

[9] Lee ZH, Avraham T, Monaco C, Patel AA, Hirsch DL, Levine JP (2018). Op**timizing functional outcomes in mandibular condyle reconstruction with the free fibula flap using computer-aided design and manufacturing technology**. J Oral Maxillofac Surg.

[10] Huys SEF, Pastor-Alonso D, Theuns P, van Lenthe  GH, Vander Sloten J, Mommaerts MY (2022). A novel 3D-printed, patient-specific alloplastic temporomandibular joint replacement allowing enthesis reconstruction: A finite element analysis. Annals of 3D Printed Medicine.

[11] Keyser B, Afzal Z, Warburton G (2020). Computer-Assisted Planning and Intraoperative Navigation in the Management of Temporomandibular Joint Ankyloses. Atlas Oral Maxillofac Surg Clin North Am.

[12] Jang BG, Huh KH,  Kang JH, Kim JE, Yi WJ, Heo MS, Lee SS (2020). Imaging features of chondrosarcoma of the temporomandibular joint: report of nine cases and literature review. Clin Radiol.

[13] Evans HL, Ayala AG, Romsdahl MM (1977). Prognostic factors in chondrosarcoma of bone: a clinicopathologic analysis with emphasis on histologic grading. Cancer.

[14] Yang HM, Kim HJ, Hu KS (2015). Anatomic and histological study of great auricular nerve and its clinical implication. J Plast Reconstr Aesthet Surg.

[15] White WM, McKenna MJ, Deschler DG (2006). Use of the thoracodorsal nerve for facial nerve grafting in the setting of pedicled latissimus dorsi reconstruction. Otolaryngol Head Neck Surg.

[16] Matsuyama T, Mackay M, Midha R (2000). Peripheral nerve repair and grafting techniques: a review. Neurol Med Chir (Tokyo).

[17] House JW, Brackmann DE (1985). Facial nerve grading system. Otolaryngol Head Neck Surg.

[18] Marx RE, Cillo JE, Broumand V, Ulloa JJ (2008). Outcome analysis of mandibular condylar replacements in tumor and trauma reconstruction: a prospective analysis of 131 cases with long-term follow-up. J Oral Maxillofac Surg.

[19] Giannakopoulos HE, Sinn DP, Quinn PD (2012). Biomet microfixation temporomandibular joint replacement system: a 3-year follow-up study of patients treated during 1995 to 2005. J Oral Maxillofac Surg.

[20] Li BH, Jung HJ, Choi SW, Kim SM, Kim MJ, Lee JH (2012). Latissimus dorsi (LD) free flap and reconstruction plate used for extensive maxillo-mandibular reconstruction after tumour ablation. J Craniomaxillofac Surg.

[21] Moher D, Liberati A, Tetzlaff J, Altman DG, Group P (2009). Preferred reporting items for systematic reviews and meta-analyses: the PRISMA statement. Ann Intern Med.

[22] Nortje CJ, Farman AG, Grotepass FW, Van Zyl JA (1976). Chondrosarcoma of the mandibular condyle. Report of a case with special reference to radiographic features. Br J Oral Surg.

[23] Looser KG, Kuehn PG (1976). Primary tumors of the mandible. A study of 49 cases. Am J Surg.

[24] Morris MR, Clark SK, Porter BA, Delbecq RJ (1987). Chondrosarcoma of the temporomandibular joint: case report. Head Neck Surg.

[25] Murayama S, Suzuki I, Nagase M, Shingaki S, Kawasaki T, Nakajima T, Fukushima M, Ishiki T (1988). Chondrosarcoma of the mandible. Report of case and a survey of 23 cases in the Japanese literature. J Craniomaxillofac Surg.

[26] Wasenko JJ, Rosenbloom SA (1990). Temporomandibular joint chondrosarcoma: CT demonstration. J Comput Assist Tomogr.

[27] Park TW, You DS, Choi SC, Lee GI (1992). Chondrosarcoma of the mandibular condyle: Report of a case. Oral Radiol.

[28] Blanchaert RH, Ord RA (1998). Vertical ramus compartment resection of the mandible for deeply invasive tumors. J Oral Maxillofac Surg.

[29] Batra PS, Estrem SA, Zitsch RP, McDonald R, Ditto J (1999). Chondrosarcoma of the temporomandibular joint. Otolaryngol Head Neck Surg.

[30] Sasaki M, Matsuda M, Ikehata M, Nara J, Kubo K, Takekawa M, Kita S (2002). Sarcomas in the Temporomandibular Joint Region: Report of 2 Cases. Asian J Oral Maxillofac Surg.

[31] Yun KI, Park MK, Kim CH, Park JU. Chondrosarcoma in the mandibular condyle : case report. J Korean Assoc Oral Maxillofac Surg. 2008;34:95–8.

[32] Acar GO, Cansiz H, Acioglu E, Mercan H, Dervisoglu S (2008). Chondrosarcoma of the mandible extending to the infratemporal fossa: report of two cases. Oral Maxillofac Surg.

[33] Gallego L, Junquera L, Fresno MF, de Vicente JC. Chondrosarcoma of the temporomandibular joint. A case report and review of the literature. Med Oral Patol Oral Cir Bucal. 2009;14:E39-43. 19114955

[34] Oliveira RC, Marques KD, Mendonca AR, Mendonca EF, Silva MR, Batista AC, Ribeiro-Rotta RF (2009). Chondrosarcoma of the temporomandibular joint: a case report in a child. J Orofac Pain.

[35] Garzino-Demo P, Tanteri G, Boffano P, Ramieri G, Pacchioni D, Maletta F, Bianchi CC, Bianchi SD, Berrone S (2010). Chondrosarcoma of the temporomandibular joint: a case report and review of the literature. J Oral Maxillofac Surg.

[36] Gonzalez-Perez LM, Sanchez-Gallego F, Perez-Ceballos JL, Lopez-Vaquero D (2011). Temporomandibular joint chondrosarcoma: Case report. J Craniomaxillofac Surg.

[37] Xu B, Shi H, Wang S, Wang P, Yu Q (2011). Secondary chondrosarcoma in the mandibular condyle. Dentomaxillofac Radiol.

[38] Deyhimi P, Keshani F (2012). Chondrosa**rcoma of the mandibular condyle: a case report**. J Dent.

[39] Ramos-Murguialday M, Lasa-Menendez V, Ignacio Iriarte-Ortabe J, Couce M (2012). Chondrosarcoma of the mandible involving angle, ramus, and condyle. J Craniofac Surg.

[40] Abu-Serriah M, Ahluwalia K, Shah KA, Bojanic S, Saeed N (2013). A novel approach to chondrosarcoma of the glenoid fossa of the temporomandibular joint: a case report. J Oral Maxillofac Surg.

[41] Goutzanis L, Kalfarentzos EF, Petsinis V, Papadogeorgakis N (2013). Chondrosarcoma of the mandibular condyle in a patient with Werner syndrome: a case report. J Craniomaxillofac Surg.

[42] Kumar Reddy DS, Kishore Kumar RV, Gali R, Kannubaddy SR, Rao M, Akheel M (2014). Central chondrosarcoma of a pediatric mandibular condy**le: a case report and review**. Ann Maxillofac Surg.

[43] Giorgione C, Passali FM, Varakliotis T, Sibilia M, Ottaviani F (2015). Temporo-mandibular joint chondrosarcoma**: case report and review of the literature**. Acta Otorhinolaryngol Ital.

[44] MacIntosh RB, Khan F, Waligora BM (2015). Chondrosarcoma of the temporomandibular disc: behavior over a 28-year observation period. J Oral Maxillofac Surg.

[45] Nomura T, Kobayashi T, Shingaki S, Saito C (2015). A case of chondrosarcoma arising in the temporomandibular joint. Case Rep Otolaryngol.

[46] Lee K, Kim SH, Kim SM, Myoung H (2016). Temporomandibular joint chondrosarcoma: a case report and literature review. J Korean Assoc Oral Maxillofac Surg.

[47] Fukada K, Okamoto T, Shibata N, Ando T (2018). A case of chondrosarcoma in the temporomandibular joint. J Oral and Maxillofac Surg Med Pathol.

[48] Inomata T, Miura K, Fushimi C, Kanno C, Kurosaka M, Matsui C (2020). Chondrosarcoma in the condyler head: A case report and review of the literature. J Oral Maxillofac Surg Med Pathol.

[49] Le QV, Nguyen DV, Nguyen HV, Hoang TD, Ngo DQ, Ngo TT (2020). Surgery and radiation management for chondrosarcoma of the temporo-mandibular join**t: a Vietnamese case report**. Int J Surg Case Rep.

[50] Ampu H, Singh T, Kumar S, Singh HP, Bhalla S: A case report of chondrosarcoma of temporomandibular joint. *Indian Journal of Otolaryngology and Head & Neck Surgery* 2021. 10.1007/s12070-021-02483-2 PMC9895524 36742594

[51] Iro S, Slimani F (2021). Diagnostic and therapeutic difficulty of chondrosarcoma of the temporomandibular joint: Two cases report. Oral and Maxillofacial Surgery Cases.

[52] Chia ZJ, Gan YJ, Lim MY (2021). Pearls on resecting the TMJ chondrosarcoma: how i do it. Eur Arch Otorhinolaryngol.

[53] Oh KY, Yoon HJ, Lee JI, Hong SP, Hong SD (2016). Chondrosarcoma of the temporomandibular joint: a case report and review of the literature. Cranio.

[54] Cohen B, Smith CJ (1963). Chondrosarcoma of the mandible. Ann R Coll Surg Engl.

[55] Molla MR, Ijuhin N, Sugata T, Sakamoto T (1987). Chondrosarcoma of the jaw: report of two cases. J Oral Maxillofac Surg.

[56] Seng C, Png W, Tan MH (2013). Accuracy of core needle biopsy for musculoskeletal tumours. J Orthop Surg (Hong Kong).

[57] Adams SC, Potter BK, Pitcher DJ, Temple HT (2010). Office-based core needle biopsy of bone and soft tissue malignancies: an accurate alternative to open biopsy with infrequent complications. Clin Orthop Relat Res.

[58] Yang CY, Wang C-P (2014). Diagnosis **of giant cell tumor of temporomandibular joint with ultrasound-guided core needle biopsy**. J Med Ultrasound.

[59] Hng J, Manchella S, Lekgabe E (2023). Gout of the temporomandibular joint and review of the literature. BJR Case Rep.

[60] Wang DD, Luo HY, Guo CB, Meng JH (2020). Clinical and immunohistochemical analysis of diffuse tenosynovial giant cell tumour of the temporomandibular joint. Int J Oral Maxillofac Surg.

[61] Carlson ER, Panella T, Holmes JD (2004). Sarcoma of mandible. J Oral Maxillofac Surg.

[62] Penel N, Van Haverbeke C, Lartigau E, Vilain MO, Van Ton J, Mallet Y, Lefebvre JL (2004). Head and neck soft tissue sarcomas of adult: prognostic value of surgery in multimodal therapeutic approach. Oral Oncol.

[63] Aziz SR, Miremadi AR, McCabe JC (2002). Mesenchymal chondrosarcoma of the maxilla with diffuse metastasis: case report and literature review. J Oral Maxillofac Surg.

[64] Gorsky M, Epstein JB (2000). Craniofacial osseous and chondromatous sarcomas in British Columbia—a review of 34 cases. Oral Oncol.

[65] de Souza LL, Pontes FSC, Fonseca FP, da Mata Rezende DS, Vasconcelos VCS, Pontes HAR: Chondrosarcoma of the jaw bones: a review of 224 cases reported to date and an analysis of prognostic factors. *Int J Oral Maxillofac Surg* 2018. 10.1016/j.ijom.2018.11.006 30528199

[66] Lee SY, Lim YC, Song MH, Seok JY, Lee WS, Choi EC (2005). Chondrosarcoma of the head and neck. Yonsei Med J.

[67] Saeed NR, Kent JN (2003). A retrospective study of the costochondral graft in TMJ reconstruction. Int J Oral Maxillofac Surg.

[68] Saeed N, Hensher R, McLeod N, Kent J (2002). Reconstruction of the temporomandibular joint autogenous compared with alloplastic. Br J Oral Maxillofac Surg.

[69] Gonzalez-Garcia R, Naval-Gias L, Rodriguez-Campo FJ, Martinez-Chacon JL, Gil-Diez Usandizaga JL (2008). Vascularized fibular flap for reconstruction of the condyle after mandibular ablation. J Oral Maxillofac Surg.

[70] Westermark A, Koppel D, Leiggener C (2006). Condylar replacement alone is not sufficient for prosthetic reconstruction of the temporomandibular joint. Int J Oral Maxillofac Surg.

[71] Carlson ER (2002). Disarticulation resections of the mandible: a prospective review of 16 cases. J Oral Maxillofac Surg.

[72] Almansoori AA, Choung HW, Kim B, Park JY, Kim SM, Lee JH (2020). Fracture o**f standard titanium mandibular reconstruction plates and preliminary study of three-dimensional printed reconstruction plates**. J Oral Maxillofac Surg.

[73] Schulz-Ertner D, Nikoghosyan A, Thilmann C, Haberer T, Jakel O, Karger C, Scholz M, Kraft G, Wannenmacher M, Debus J: Carbon ion radiotherapy for chordomas and low-grade chondrosarcomas of the skull base. Results in 67 patients. *Strahlenther Onkol* 2003, 179:598–605. 10.1007/s00066-003-1120-2 14628125

[74] Noel G, Habrand JL, Jauffret E, de Crevoisier R, Dederke S, Mammar H, Haie-Meder C, Pontvert D, Hasboun D, Ferrand R (2003). Radiation therapy for chordoma and chondrosarcoma of the skull base and the cervical spine. Prognostic factors and patterns of failure. Strahlenther Onkol.

[75] Ruark DS, Schlehaider UK, Shah JP (1992). Chondrosarcomas of the head and neck. World J Surg.

[76] Burkey BB, Hoffman HT, Baker SR, Thornton AF, McClatchey KD (1990). Chondrosarcoma of the head and neck. Laryngoscope.

[77] McNaney D, Lindberg RD, Ayala AG, Barkley HT, Hussey DH (1982). Fifteen year radiotherapy experience with chondrosarcoma of bone. Int J Radiat Oncol Biol Phys.

[78] Thorkildsen J, Taksdal I, Bjerkehagen B, Haugland HK, Borge Johannesen T, Viset T, Norum OJ, Bruland O, Zaikova O: Chondrosarcoma in Norway 1990–2013; an epidemiological and prognostic observational study of a complete national cohort. *Acta Oncol* 2019:1–10. 10.1080/0284186X.2018.1554260 30632866

[79] Ichikawa T, Miyauchi M, Nikai H, Yoshiga K (1998). Synovial chondrosarcoma arising in the temporomandibular joint. J Oral Maxillofac Surg.

